# Simple psychiatric screening for burning mouth syndrome and persistent globus pharyngeus using the hospital anxiety and depression scale: a case-control study

**DOI:** 10.1016/j.bjorl.2026.101940

**Published:** 2026-11-06

**Authors:** Hong Kwon Kil, Hyun-Ju Kim, Min-Su Kim

**Affiliations:** a Department of Otorhinolaryngology-Head and Neck Surgery, CHA Bundang Medical Center, CHA University School of Medicine, Seongnam, Republic of Korea; b Department of Psychiatry, CHA Bundang Medical Center, CHA University School of Medicine, Seongnam, Republic of Korea

**Keywords:** Burning mouth syndrome, Gastro-esophageal reflux, Anxiety, Depression, Psychological assessment

## Abstract

**Objective:**

To evaluate a simple psychiatric screening approach for functional otorhinolaryngologic disorders ‒ conditions that show no abnormalities on standard work-up and respond poorly to medication ‒ we analyzed Hospital Anxiety and Depression Scale (HADS) results in patients with Burning Mouth Syndrome (BMS) and persistent Globus Pharyngeus (GP) commonly seen in otorhinolaryngologic clinic, where psychiatric assessment is unfamiliar to most otorhinolaryngologists.

**Methods:**

We conducted a single-center, retrospective case-control study including 185 subjects with BMS (n = 54), persistent GP (n = 69) (persisting ≥3-month despite anti-reflux medication), and controls (n = 62). Psychiatric screening used the HADS with a prespecified cut-off of ≥8 to define abnormal scores (0–7 normal, 8–10 mild, 11–14 moderate, 15–21 severe). Primary outcomes were abnormal anxiety and depression on HADS. We used logistic regression adjusted for age, sex, and prespecified predictors to estimate adjusted Odds Ratios (aOR) with 95% Confidence Intervals (95% CIs).

**Results:**

Patients with moderate-to-severe HADS scores in anxiety or depression (47/185, 25.4%) all accepted psychiatric consultation (47/47, 100%). Abnormal anxiety occurred in 37.0% of BMS (20/54), 46.4% of GP (32/69), and 11.3% of controls (7/62); abnormal depression in 51.9% of BMS (28/54), 55.1% of GP (38/69), and 17.7% of controls (11/62), respectively. Compared with controls, odds of anxiety and depression were higher in both patient groups: [BMS ‒ anxiety (aOR = 4.254; 95% CI 1.606–11.269), depression (aOR = 4.512; 95% CI 1.902–10.701)] and [GP ‒ anxiety (aOR = 6.827; 95% CI 2.710–17.197), depression (aOR = 5.743; 95% CI 2.545–12.961)].

**Conclusion:**

Patients with BMS or persistent GP had higher levels of anxiety and depression than controls. A brief HADS-based screening proved clinically feasible in an otorhinolaryngology setting by identifying patients with substantial psychiatric burden and facilitating acceptance of psychiatric referral among moderate-to-severe cases. These findings may support the integration of HADS-guided psychiatric screening and referral into routine otorhinolaryngologic care.

**Level of evidence:**

Level 4.

## Highlights

• Both BMS and persistent GP showed higher anxiety and depression than controls.
• Cancer survivorship was associated with higher anxiety and depression in BMS.
• All patients with moderate-to-severe HADS scores accepted psychiatric referral.
• HADS enables brief psychiatric screening for BMS and persistent GP in practice.
• HADS-guided screening/referral may be integrated into other functional ENT disorders.

## Introduction

Many otorhinolaryngologists frequently encounter patients whose symptoms show no abnormalities on standard work-up and respond poorly to empirical medication. These functional otorhinolaryngologic disorders often have psychological components.^1^

BMS is a chronic condition characterised by intraoral burning sensations without identifiable local or systemic causes. A systematic review and meta-analysis reported an overall pooled prevalence of 1.73% (95% Conﬁdence Interval [95% CI 0.176–0.351], n = 26,632) in the general population and 7.72% (95% CI 0.434–0.691, n = 86,591) in dental clinic patients.^2^ Despite advances in treatment, BMS remains challenging; some patients experience partial remission, but many continue to have persistent symptoms and no definitive therapy is available.^3^ Clonazepam appears to be one of the more effective drugs, with a response rate of approximately 60%.^4^ Although its exact etiology remains unclear, psychological factors ‒ particularly anxiety and depression ‒ play a crucial role in the manifestation and persistence of BMS.^5^^,^^6^ Recent studies underline this psychosocial burden; for example, in a Japanese series, 51.8% (43/83) of BMS patients had comorbid psychiatric disorders.^7^

GP is characterized by a sense of tightness or a non-painful lump or foreign-body sensation in the throat. Historically, it was regarded as psychogenic and termed “globus hystericus”, especially in anxious women, but this term was later replaced by GP when Malcomson recognized that not all patients were hysterical or female.^8^ Laryngopharyngeal Reflux (LPR) is frequently implicated; in one survey, up to 48% of otorhinolaryngologists considered LPR to be highly related to GP.^9^ Proton Pump Inhibitors (PPIs) are commonly used to manage suspected LPR, yet a systematic review including two systematic reviews and seven meta-analyses concluded that the benefit of PPI therapy for LPR remains uncertain: most reviews found PPIs no better than placebo, and those reporting symptom improvement did not show clear endoscopic changes.^10^ Overall, GP is now considered a multifactorial condition in which psychological factors are central, especially in persistent cases. In a Chinese cohort of 619 patients, those with persistent GP had significantly higher rates of anxiety (69.8% vs. 45.74%, p < 0.001) and depression (48.99% vs. 21.06%, p < 0.001) than those with non- persistent GP.^11^

Given this background, psychiatric evaluation is important when assessing functional otorhinolaryngologic disorders. Nevertheless, routine psychiatric screening is rarely implemented in busy otorhinolaryngologic clinics, and such assessment can be unfamiliar and challenging for many otorhinolaryngologists. The Hospital Anxiety and Depression Scale (HADS) is a widely used, validated tool for screening both anxiety and depression, particularly in patients with somatic comorbidities.^12^, ^13^, ^14^, ^15^ Zigmond and Snaith originally introduced HADS, emphasizing its suitability for non-psychiatric clinical settings owing to its relatively short number of questions and simple interpretation.^16^ Additionally, prior research found no significant difference in screening performance between HADS, the Beck Depression Inventory, and the Hamilton Depression Rating Scale in stroke patients.^17^ A review by Bjelland et al. confirmed the validity and reliability of HADS and highlighted its high acceptability, brevity (2–6 min), and ease of interpretation, facilitating use by non-psychiatrists.^18^ Consequently, HADS is well suited for use by otorhinolaryngologists to screen for anxiety and depression in functional disorders.

Therefore, we evaluated anxiety and depression using HADS in two prototypical functional otorhinolaryngologic disorders commonly seen in outpatient practice ‒ BMS and persistent GP ‒ comparing each with controls and exploring within-group clinical correlates.

## Methods

### Study population

This single-center study was designed as a retrospective case-control study utilizing data collected as part of a routine clinical protocol. At our institution, the HADS was administered as part of routine care at the time of visit for adult patients. We retrospectively reviewed these routinely collected HADS data and the related medical records of consecutive patients diagnosed with BMS or persistent GP between January and October 2024. The study was approved by the institutional review board and conducted in accordance with the Declaration of Helsinki; informed consent was waived due to the retrospective nature of the study.

We included and excluded the following categories of patients with BMS ‒Inclusion: (1) Daily intraoral burning/dysesthesia >2-hs/day for >3-months; (2) No causative lesions on otorhinolaryngologic examination; (3) Normal screening laboratories ‒ Exclusion: (1) Symptoms secondary to otorhinolaryngology-head and neck surgery; (2) Prior psychiatric diagnosis or current antidepressant/antipsychotic/antiepileptic therapy; (3) <5-years since treatment for any cancer.

We included and excluded the following categories of patients with persistent GP ‒ Inclusion: (1) Persistent globus despite ≥3-month of proton-pump inhibitor or potassium-competitive acid blocker therapy; (2) no gross pharyngo-laryngeal lesion; (3) Normal screening laboratories ‒ Exclusion: (1) Symptoms secondary to otorhinolaryngology-head and neck surgery; (2) Prior psychiatric diagnosis or current antidepressant/antipsychotic/antiepileptic therapy; (3) <5-years since treatment for any cancer.

All patients underwent history taking, physical examination, laryngoscopy, and screening tests (laboratory panels or neck/thyroid ultrasonography). When no abnormalities were identified, HADS was administered.

Controls without BMS or persistent GP were selected from our department patients with mild otorhinolaryngologic conditions (e.g., allergic rhinitis, acute sinusitis, mild sensorineural hearing loss, or mild snoring) who had also undergone HADS assessment as part of the same routine clinical protocol. Their medical records were retrospectively reviewed to establish a comparison group. To minimize potential confounding factors, we performed a case-control matching process focusing on age, sex, and comorbidities, which were identified as potential biases in the preliminary analysis.

Recorded covariates were age, sex, hypertension, diabetes mellitus, hyperlipidemia, and cancer survivorship; hypertension/diabetes mellitus/hyperlipidemia were defined by use of disease-specific medications.

### HADS

HADS, developed by Zigmond and Snaith, is a simple one-page test consisting of 14 questions. The HADS is a highly reliable (Cronbach’s alpha = 0.80‒0.93 for the anxiety subscale, 0.81‒0.90 for the depression subscale) and highly valid assessment.^19^ The assessment is divided into two subscales: one with seven items to assess anxiety and seven items to evaluate depression. The scores for each question range from 0 to 3. Thus, the total score for each subscale (seven questions) ranges from 0 to 21. If the scores are ≥8 for either question set, abnormal anxiety or depression is suspected. In addition, based on the total scores in each subscale, the levels of anxiety and depression were defined as follows: 0–7 = normal, 8–10 = mild, 11–14 = moderate, and 15–21 = severe.

### Statistical analysis

Continuous variables are presented as mean ± SD and compared with *t*-tests/ANOVA after normality checks; categorical variables as n (%) and compared with χ^2^ or Fisher’s exact tests. Linear-by-linear association tested ordinal HADS grades. Primary outcomes were abnormal anxiety and depression in HADS (cut-off ≥8). For between-group comparisons (BMS vs. controls; persistent GP vs. controls), logistic regression was used to estimate adjusted Odds Ratios (aORs) with 95% CIs. Age and sex were forced into all models; additionally, binary covariates (hypertension, diabetes mellitus, hyperlipidemia, cancer survivorship) showing univariable p < 0.05 for the outcome were included. The same approach was applied to within-group analyses in BMS and persistent GP. Where zero cell counts caused complete separation, effect sizes were reported as non-estimable with the Fisher's exact test p-value. Two-sided α = 0.05 defined significance. Analyses used IBM SPSS Statistics, version 22 (IBM Corp., Armonk, NY, USA).

## Results

Baseline characteristics of 185 participants ‒ controls (n = 62), BMS (n = 54), persistent GP (n = 69) ‒ are presented in Table 1. Patients with moderate-to-severe HADS scores in anxiety or depression (47/185, 25.4%) all accepted psychiatric consultation (47/47, 100%). Compared with controls, BMS showed higher rates of abnormal anxiety (37.0% vs. 11.3%; aOR = 4.254; 95% CI 1.606–11.269) and depression (51.9% vs. 17.7%; aOR = 4.512; 95% CI 1.902–10.701) (Table 2). In BMS, cancer survivorship independently predicted abnormal anxiety (aOR = 15.694; 95% CI 1.399–176.076). For depression, cancer survivorship was significantly associated with depression level (p = 0.024); however, standard logistic regression analysis could not estimate OR and aOR owing to zero events in the normal subgroup. Other covariates (age, sex, hypertension, diabetes, and hyperlipidemia) were not associated (Table 3). Compared with controls, persistent GP likewise had higher abnormal anxiety (46.4% vs. 11.3%; aOR = 6.827; 95% CI 2.710–17.197) and depression (55.1% vs. 17.7%; aOR = 5.743; 95% CI 2.545–12.961) (Table 4). In persistent GP, no independent risk factors were identified after adjustment (e.g., hypertension: aOR = 0.313; 95% CI 0.071–1.373; non-significant) (Table 5). Psychiatric burden did not differ significantly between BMS and persistent GP in the abnormal anxiety (37.0% vs. 46.4%; aOR = 1.470; 95% CI 0.697–3.098) or abnormal depression (51.9% vs. 55.1%; aOR = 1.133; 95% CI 0.546–2.350) (Table 6).

**Table 1:** Baseline characteristics of the study population (n = 185): controls (n = 62), burning mouth syndrome (n = 54), and persistent globus pharyngeus (n = 69).

Variables	Control (n = 62)	BMS (n = 54)	Globus (n = 69)	Statistics
	n (%) or mean ± SD	n (%) or mean ± SD	n (%) or mean ± SD	p-value
Age (years)	51.1 ± 17.0	55.7 ± 16.3	51.6 ± 14.9	0.244
Sex				0.464
Male	25 (40.3%)	16 (29.6%)	23 (33.3%)	
Female	37 (59.7%)	38 (70.4%)	46 (66.7%)	
Hypertension				0.416
No	44 (71.0%)	43 (79.6%)	55 (79.7%)	
Yes	18 (29.0%)	11 (20.4%)	14 (20.3%)	
Diabetes				0.604
No	57 (91.9%)	52 (96.3%)	64 (92.8%)	
Yes	5 (8.1%)	2 (3.7%)	5 (7.2%)	
Hyperlipidemia				0.308
No	53 (85.5%)	40 (74.1%)	55 (79.7%)	
Yes	9 (14.5%)	14 (25.9%)	14 (20.3%)	
Cancer survivor				0.417
No	59 (95.2%)	48 (88.9%)	62 (89.9%)	
Yes	3 (4.8%)	6 (11.1%)	7 (10.1%)	
Anxiety subscale	4.2 ± 3.3	6.8 ± 4.2	7.2 ± 4.2	<0.001^a^
2 groups				<0.001^a^
Normal (0–7)	55 (88.7%)	34 (63.0%)	37 (53.6%)	
Abnormal (8–21)	7 (11.3%)	20 (37.0%)	32 (46.4%)	
4 groups				0.002^a^
Normal (0–7)	55 (88.7%)	34 (63.0%)	37 (53.6%)	
Mild (8–10)	5 (8.1%)	10 (18.5%)	19 (27.5%)	
Moderate (11–14)	1 (1.6%)	8 (14.8%)	9 (13.0%)	
Severe (15–21)	1 (1.6%)	2 (3.7%)	4 (5.8%)	
Depression subscale	5.4 ± 3.7	7.2 ± 4.0	7.9 ± 4.1	0.001^a^
2 groups				<0.001^a^
Normal (0–7)	51 (82.3%)	26 (48.1%)	31 (44.9%)	
Abnormal (8–21)	11 (17.7%)	28 (51.9%)	38 (55.1%)	
4 groups				<0.001^a^
Normal (0‒7)	51 (82.3%)	26 (48.1%)	31 (44.9%)	
Mild (8–10)	5 (8.1%)	18 (33.3%)	19 (27.5%)	
Moderate (11–14)	4 (6.5%)	9 (16.7%)	17 (24.6%)	
Severe (15–21)	2 (3.2%)	1 (1.9%)	2 (2.9%)	

n, Number; SD, Standard Deviation; BMS, Burning Mouth Syndrome.

a Statistically significant.

**Table 2:** Baseline characteristics and psychiatric outcomes in burning mouth syndrome vs. controls.

Variables	Control (n = 62)	BMS (n = 54)	Statistics		
	n (%) or mean ± SD	n (%) or mean ± SD	p-value	OR (95% CI)	aOR (95% CI)
Age (years)	51.1 ± 17.0	55.7 ± 16.3	0.140	N/A	
Sex			0.229	1.605 (0.740–3.479)	
Male	25 (40.3%)	16 (29.6%)			
Female	37 (59.7%)	38 (70.4%)			
HTN			0.283	0.625 (0.265–1.477)	
No	44 (71.0%)	43 (79.6%)			
Yes	18 (29.0%)	11 (20.4%)			
DM			0.477	0.438 (0.082–2.358)	
No	57 (91.9%)	52 (96.3%)			
Yes	5 (8.1%)	2 (3.7%)			
HL			0.124	2.061 (0.811–5.237)	
No	53 (85.5%)	40 (74.1%)			
Yes	9 (14.5%)	14 (25.9%)			
Ca survivor			0.300	2.458 (0.584–10.348)	
No	59 (95.2%)	48 (88.9%)			
Yes	3 (4.8%)	6 (11.1%)			
Anxiety subscale	4.2 ± 3.3	6.8 ± 4.2	<0.001^a^	N/A	
2 groups			0.001^a^	4.622^a^ (1.768‒12.084)	4.254^a^ (1.606‒11.269)
Normal (0–7)	55 (88.7%)	34 (63.0%)			
Abnormal (8–21)	7 (11.3%)	20 (37.0%)			
4 groups			0.002^a^	N/A	
Normal (0–7)	55 (88.7%)	34 (63.0%)			
Mild (8–10)	5 (8.1%)	10 (18.5%)			
Moderate (11‒14)	1 (1.6%)	8 (14.8%)			
Severe (15–21)	1 (1.6%)	2 (3.7%)			
Depression subscale	5.4 ± 3.7	7.2 ± 4.0	0.013^a^	N/A	
2 groups			<0.001^a^	4.993^a^ (2.151‒11.592)	4.512^a^ (1.902‒10.701)
Normal (0–7)	51 (82.3%)	26 (48.1%)			
Abnormal (8–21)	11 (17.7%)	28 (51.9%)			
4 groups			0.005^a^	N/A	
Normal (0–7)	51 (82.3%)	26 (48.1%)			
Mild (8–10)	5 (8.1%)	18 (33.3%)			
Moderate (11–14)	4 (6.5%)	9 (16.7%)			
Severe (15–21)	2 (3.2%)	1 (1.9%)			

n, Number; SD, Standard Deviation; BMS, Burning Mouth Syndrome; OR, Odds Ratio; CI, Confidence Interval; aOR, adjusted Odds Ratio; N/A, Not Applicable; HTN, Hypertension; DM, Diabetes Mellitus; HL, Hyperlipidemia; Ca, Cancer.

a Statistically significant.

**Table 3:** Risk factors for anxiety and depression within patients with burning mouth syndrome.

Anxiety (HADS-A)					
	Normal (n = 34)	Abnormal (n = 20)	Statistics		
	n (%) or mean ± SD	n (%) or mean ± SD	p‒value	OR (95% CI)	aOR (95% CI)
Age (years)	55.8 ± 16.0	55.6 ± 17.2	0.959	N/A	
Scale				N/A	
Anxiety	4.0 ± 1.8	11.6 ± 2.5	<0.001^a^		
Depression	5.4 ± 3.2	10.2 ± 3.4	<0.001^a^		
Sex			0.964	0.972 (0.291–3.253)	
Male	10 (29.4%)	6 (30.0%)			
Female	24 (70.6%)	14 (70.0%)			
HTN			0.072	0.126 (0.015–1.076)	
No	24 (70.6%)	19 (95.0%)			
Yes	10 (29.4%)	1 (5.0%)			
DM			0.525	N/A	
No	32 (94.1%)	20 (100.0%)			
Yes	2 (5.9%)	0 (0.0%)			
HL			0.243	2.077 (0.602–7.170)	
No	27 (79.4%)	13 (65.0%)			
Yes	7 (20.6%)	7 (35.0%)			
Ca survivorship			0.022^a^	11.000^a^ (1.180‒102.513)	15.694^a^ (1.399‒176.076)
No	33 (97.1%)	15 (75.0%)			
Yes	1 (2.9%)	5 (25.0%)			

Depression (HADS-D)					
	Normal (n = 26)	Abnormal (n = 28)	Statistics		
	n (%) or mean ± SD	n (%) or mean ± SD	p‒value	OR (95% CI)	aOR (95% CI)
Age (years)	55.0 ± 16.3	56.4 ± 16.5	0.763	N/A	
Scale				N/A	
Anxiety	4.4 ± 3.0	9.1 ± 3.9	<0.001^a^		
Depression	3.7 ± 1.8	10.5 ± 2.3	<0.001^a^		
Sex			0.675	0.778 (0.240–2.518)	
Male	7 (26.9%)	9 (32.1%)			
Female	19 (73.1%)	19 (67.9%)			
HTN			0.320	0.452 (0.115–1.777)	
No	19 (73.1%)	24 (85.7%)			
Yes	7 (26.9%)	4 (14.3%)			
DM			1.000	0.926 (0.055–15.607)	
No	25 (96.2%)	27 (96.4%)			
Yes	1 (3.8%)	1 (3.6%)			
HL			0.358	1.989 (0.566–6.993)	
No	21 (80.8%)	19 (67.9%)			
Yes	5 (19.2%)	9 (32.1%)			
Ca survivorship			0.024^a^	N/A	N/A
No	26 (100.0%)	22 (78.6%)			
Yes	0 (0.0%)	6 (21.4%)			

n, number; SD, Standard Deviation; HADS, Hospital Anxiety Depression Scale; OR, Odds Ratio; CI, Confidence Interval; aOR, adjusted Odds Ratio; N/A, Not Applicable; HTN, Hypertension; DM, Diabetes Mellitus; HL, Hyperlipidemia; Ca, Cancer.

a Statistically significant.

**Table 4:** Baseline characteristics and psychiatric outcomes in persistent globus pharyngeus vs. controls.

Variables	Control (n = 62)	Globus (n = 69)	Statistics		
	n (%) or mean ± SD	n (%) or mean ± SD	p-value	OR (95% CI)	aOR (95% CI)
Age (years)	51.1 ± 17.0	51.6 ± 14.9	0.854	N/A	
Sex			0.407	1.351 (0.663–2.756)	
Male	25 (40.3%)	23 (33.3%)			
Female	37 (59.7%)	46 (66.7%)			
HTN			0.245	0.622 (0.279–1.389)	
No	44 (71.0%)	55 (79.7%)			
Yes	18 (29.0%)	14 (20.3%)			
DM			0.860	0.891 (0.245–3.235)	
No	57 (91.9%)	64 (92.8%)			
Yes	5 (8.1%)	5 (7.2%)			
HL			0.386	1.499 (0.598–3.756)	
No	53 (85.5%)	55 (79.7%)			
Yes	9 (14.5%)	14 (20.3%)			
Ca survivor			0.332	2.220 (0.548–8.993)	
No	59 (95.2%)	62 (89.9%)			
Yes	3 (4.8%)	7 (10.1%)			
Anxiety subscale	4.2 ± 3.3	7.2 ± 4.2	<0.001^a^	N/A	
2 groups			<0.001^a^	6.795^a^ (2.714‒17.015)	6.827^a^ (2.710‒17.197)
Normal (0–7)	55 (88.7%)	37 (53.6%)			
Abnormal (8–21)	7 (11.3%)	32 (46.4%)			
4 groups			<0.001^a^	N/A	
Normal (0–7)	55 (88.7%)	37 (53.6%)			
Mild (8–10)	5 (8.1%)	19 (27.5%)			
Moderate (11–14)	1 (1.6%)	9 (13.0%)			
Severe (15–21)	1 (1.6%)	4 (5.8%)			
Depression subscale	5.4 ± 3.7	7.9 ± 4.1	<0.001^a^	N/A	
2 groups			<0.001^a^	5.683^a^ (2.539‒12.724)	5.743^a^ (2.545‒12.961)
Normal (0–7)	51 (82.3%)	31 (44.9%)			
Abnormal (8–21)	11 (17.7%)	38 (55.1%)			
4 groups			<0.001^a^	N/A	
Normal (0‒7)	51 (82.3%)	31 (44.9%)			
Mild (8–10)	5 (8.1%)	19 (27.5%)			
Moderate (11–14)	4 (6.5%)	17 (24.6%)			
Severe (15–21)	2 (3.2%)	2 (2.9%)			

n, number; SD, Standard Deviation; OR, Odds Ratio; CI, Confidence Interval; aOR, adjusted Odds Ratio; N/A, Not Applicable; HTN, Hypertension; DM, Diabetes Mellitus; HL, Hyperlipidemia; Ca, Cancer.

a Statistically significant.

**Table 5:** Risk factors for anxiety and depression within patients with persistent globus pharyngeus.

Anxiety (HADS-A)					
	Normal (n = 37)	Abnormal (n = 32)	Statistics		
	n (%) or mean ± SD	n (%) or mean ± SD	p‒value	OR (95% CI)	aOR (95% CI)
Age (years)	54.4 ± 14.6	48.3 ± 14.7	0.090	N/A	
Scale				N/A	
Anxiety	4.0 ± 2.2	10.9 ± 2.6	<0.001^a^		
Depression	5.8 ± 3.6	10.3 ± 3.2	<0.001^a^		
Sex			0.864	0.916 (0.336–2.498)	
Male	12 (32.4%)	11 (34.4%)			
Female	25 (67.6%)	21 (65.6%)			
HTN			0.042^a^	0.245^a^ (0.061‒0.974)	0.313 (0.071–1.373)
No	26 (70.3%)	29 (90.6%)			
Yes	11 (29.7%)	3 (9.4%)			
DM			0.363	0.266 (0.028–2.514)	
No	33 (89.2%)	31 (96.9%)			
Yes	4 (10.8%)	1 (3.1%)			
HL			0.366	1.722 (0.527–5.633)	
No	31 (83.8%)	24 (75.0%)			
Yes	6 (16.2%)	8 (25.0%)			
Ca survivorship			1.000	0.853 (0.176–4.135)	
No	33 (89.2%)	29 (90.6%)			
Yes	4 (10.8%)	3 (9.4%)			

Depression (HADS-D)					
	Normal (n = 31)	Abnormal (n = 38)	Statistics		
	n (%) or mean ± SD	n (%) or mean ± SD	p-value	OR (95% CI)	aOR (95% CI)
Age (years)	53.3 ± 16.3	50.2 ± 13.7	0.391	N/A	
Scale				N/A	
Anxiety	5.2 ± 3.8	8.8 ± 3.8	<0.001^a^		
Depression	4.1 ± 2.4	10.9 ± 2.2	<0.001^a^		
Sex			0.231	0.533 (0.190–1.501)	
Male	8 (25.8%)	15 (39.5%)			
Female	23 (74.2%)	23 (60.5%)			
HTN			0.136	0.370 (0.109–1.253)	
No	22 (71.0%)	33 (86.8%)			
Yes	9 (29.0%)	5 (13.2%)			
DM			0.651	0.519 (0.081–3.318)	
No	28 (90.3%)	36 (94.7%)			
Yes	3 (9.7%)	2 (5.3%)			
HL			0.669	0.774 (0.239–2.508)	
No	24 (77.4%)	31 (81.6%)			
Yes	7 (22.6%)	7 (18.4%)			
Ca survivorship			1.000	1.098 (0.227–5.322)	
No	28 (90.3%)	34 (89.5%)			
Yes	3 (9.7%)	4 (10.5%)			

n, number; SD, Standard Deviation; HADS, Hospital Anxiety Depression Scale; OR, Odds Ratio; CI, Confidence Interval; aOR, adjusted Odds Ratio; N/A, Not Applicable; HTN, Hypertension; DM, Diabetes Mellitus; HL, Hyperlipidemia; Ca, Cancer.

a Statistically significant.

**Table 6:** Baseline characteristics and psychiatric outcomes in burning mouth syndrome vs. persistent globus pharyngeus.

Variables	BMS (n = 54)	Globus (n = 69)	Statistics		
	n (%) or mean ± SD	n (%) or mean ± SD	p-value	OR (95% CI)	aOR (95% CI)
Age (years)	55.7 ± 16.3	51.6 ± 14.9	0.149	N/A	
Sex			0.661	0.842 (0.390–1.817)	
Male	16 (29.6%)	23 (33.3%)			
Female	38 (70.4%)	46 (66.7%)			
HTN			0.991	0.995 (0.411–2.410)	
No	43 (79.6%)	55 (79.7%)			
Yes	11 (20.4%)	14 (20.3%)			
DM			0.465	2.031 (0.379–10.900)	
No	52 (96.3%)	64 (92.8%)			
Yes	2 (3.7%)	5 (7.2%)			
HL			0.459	0.727 (0.312–1.694)	
No	40 (74.1%)	55 (79.7%)			
Yes	14 (25.9%)	14 (20.3%)			
Ca survivor			0.863	0.903 (0.285–2.863)	
No	48 (88.9%)	62 (89.9%)			
Yes	6 (11.1%)	7 (10.1%)			
Anxiety subscale	6.8 ± 4.2	7.2 ± 4.2	0.628	N/A	
2 groups			0.298	1.470 (0.710–3.043)	1.470 (0.697–3.098)
Normal (0–7)	34 (63.0%)	37 (53.6%)			
Abnormal (8–21)	20 (37.0%)	32 (46.4%)			
4 groups			0.470	N/A	
Normal (0–7)	34 (63.0%)	37 (53.6%)			
Mild (8–10)	10 (18.5%)	19 (27.5%)			
Moderate (11–14)	8 (14.8%)	9 (13.0%)			
Severe (15–21)	2 (3.7%)	4 (5.8%)			
Depression subscale	7.2 ± 4.0	7.9 ± 4.1	0.363	N/A	
2 groups			0.722	1.138 (0.557–2.325)	1.133 (0.546–2.350)
Normal (0–7)	26 (48.1%)	31 (44.9%)			
Abnormal (8–21)	28 (51.9%)	38 (55.1%)			
4 groups			0.394	N/A	
Normal (0–7)	26 (48.1%)	31 (44.9%)			
Mild (8–10)	18 (33.3%)	19 (27.5%)			
Moderate (11–14)	9 (16.7%)	17 (24.6%)			
Severe (15–21)	1 (1.9%)	2 (2.9%)			

n, Number; SD, Standard Deviation; OR, Odds Ratio; CI, Confidence Interval; aOR, adjusted Odds Ratio; N/A, Not Applicable; HTN, Hypertension; DM, Diabetes Mellitus; HL, Hyperlipidemia; Ca, Cancer.

## Discussion

In our study, all patients with moderate-to-severe HADS subscale scores in anxiety or depression accepted psychiatric consultation (100%), supporting feasibility in routine practice. Patients with BMS or persistent GP may resist psychiatric referral when it is suggested without an objective psychiatric screening. However, many otorhinolaryngologists have been unfamiliar with formal psychiatric assessments. A brief, clinic-ready tool such as HADS can therefore function as a bridge. It is worth noting that this high acceptance rate may be partially attributed to the accessibility of the South Korean healthcare system. South Korea offers high accessibility to specialist care with relatively low out-of-pocket costs under the National Health Insurance service. This structural environment likely facilitated the logistics of consultation. However, given that mental health stigma remains a significant cultural barrier in South Korean society, financial accessibility alone does not explain the universal acceptance. In this context, the objective data provided by HADS likely played a pivotal role in convincing patients that their symptoms warranted medical attention, thereby helping them overcome cultural resistance to psychiatric care.

According to the National Institute for Health and Care Excellence (NICE) guideline, individuals with mild HADS scores (8–10) can benefit from exercise, cognitive-behavioral therapy, or problem-solving strategies; those with moderate-to-severe HADS scores (≥11) merit psychiatric evaluation for active treatment by psychiatrists. Thus, otorhinolaryngologists would recommend consultation with psychiatrists for effective and active intervention for those with moderate-to-severe HADS subscale scores (≥11).^20^^,^^21^ Routine HADS screening followed by threshold-based triage can streamline care for these patients. In addition, combining psychological interventions (e.g., cognitive-behavioral therapy and psychological medication) with conventional care may offer more comprehensive management.

In Korea’s 2021 national survey (n = 5,511), the lifetime prevalences of anxiety and depressive disorders were 9.3% and 7.7%, with 12-month prevalences of 3.1% and 1.7%, respectively.^22^ During the COVID-19 pandemic, a population survey (n = 549) reported anxiety and depression symptoms in 10.6% and 8.8%.^23^ In a meta-analysis of 208 studies in head and neck cancer (n = 654 ,413), 17.8% reported anxiety and 19.5% reported depression.^24^ In our cohort, patients with BMS and persistent GP showed higher burdens of anxiety (37.0% and 46.4%) and depression (51.9% and 55.1%) than the general population, COVID-19 cohorts, and even head and neck cancer populations, underscoring the psychological load of these functional otorhinolaryngologic disorders.

Among patients with BMS, cancer survivorship was associated with greater anxiety (with signals for depression), consistent with literature showing elevated anxiety/depression in cancer survivors.^24^^,^^25^ A meta-analysis also showed high prevalences of anxiety (10%) and depression (15 %–20 %) in patients cured of cancer.^26^ We excluded patients undergoing active cancer treatment and included only those > 5 years post-treatment without recurrence. Nevertheless, cancer survivorship appeared to impact mental health in the long term. Thus, targeted psychiatric screening is warranted for BMS patients with a history of cancer survivorship.

This study has some limitations. First, the control group consisted of patients with mild otorhinolaryngologic conditions rather than healthy volunteers. Second, it is a retrospective, single-center analysis with a modest sample; HADS is a self-report instrument subject to acquiescence bias; subsequent psychiatric diagnoses were not verified; and post-referral symptom trajectories were not assessed. Therefore, prospective, multi-center studies are needed, followed by the development of standardized protocols that integrate psychological screening into routine practice for patients with BMS and persistent GP. Third, we did not evaluate post-referral treatment response; thus, our findings support feasibility of screening-and-referral rather than therapeutic efficacy.

## Conclusion

Compared with controls, both patients with BMS and persistent GP showed significantly higher levels of anxiety and depression. In addition, cancer survivorship was associated with an increased risk of anxiety and depression among patients with BMS. Patients with moderate-to-severe HADS scores accepted psychiatric referral. This study demonstrates that a brief HADS-based psychiatric screening approach, administered by otorhinolaryngologists, is clinically feasible for identifying psychiatric burden in patients with BMS and persistent GP. These findings may support the need to incorporate HADS-guided psychiatric screening and referral into routine otorhinolaryngologic practice and provide a basis for extending this approach to other functional otorhinolaryngologic disorders.

## ORCID ID

Hong Kwon Kil: 0000-0003-3895-3299

Hyun-Ju Kim: 0000-0002-8093-3366

Min-Su Kim: 0000-0001-8311-4174

## Authors’ contributions

Conception and design: M-S. Kim. Analysis and interpretation of the data: HK. Kil, H-J Kim, and M-S. Kim. Drafting of the article: H-J Kim and M-S. Kim. Critical revision of the article: HK. Kil, H-J Kim, and M-S. Kim. Final approval of the article: M-S. Kim. Administrative, technical, or logistic support: M-S. Kim.

## Funding and conflicts of interest

This research was supported by a grant from the Korea Health Technology R&D Project through the Korea Health Industry Development Institute (KHIDI), funded by the Ministry of Health & Welfare, Republic of Korea (Grant nº RS-2024-00439906). No potential conflict of interest relevant to this article was reported.

## Data availability statement

All data can be provided upon request to the corresponding author.

## References

[1] Al-Rawashdeh B.M., Saleh M.Y.N., Mustafa R.B. (2019). Prevalence of depression and anxiety among otolaryngology outpatients at Jordan University Hospital. Perspect Psychiatr Care..

[2] Wu S., Zhang W., Yan J., Noma N., Young A., Yan Z. (2022). Worldwide prevalence estimates of burning mouth syndrome: A systematic review and meta-analysis. Oral Dis..

[3] Klein B., Thoppay J.R., De Rossi S.S., Ciarrocca K. (2020). Burning mouth syndrome. Dermatol Clin..

[4] Liu Y.F., Kim Y., Yoo T., Han P., Inman J.C. (2018). Burning mouth syndrome: a systematic review of treatments. Oral Dis..

[5] Schiavone V., Adamo D., Ventrella G. (2012). Anxiety, depression, and pain in burning mouth syndrome: first chicken or egg?. Headache..

[6] Kim J.Y., Kim Y.S., Ko I., Kim D.K. (2020). Association Between Burning Mouth Syndrome and the Development of Depression, Anxiety, Dementia, and Parkinson Disease. JAMA Otolaryngol Head Neck Surg..

[7] Tu T.T.H., Miura A., Shinohara Y. (2018). Evaluating burning mouth syndrome as a comorbidity of atypical odontalgia: the impact on pain experiences. Pain Pract..

[8] Järvenpää P., Arkkila P., Aaltonen L.M. (2018). Globus pharyngeus: a review of etiology, diagnostics, and treatment. Eur Arch Otorhinolaryngol..

[9] Ford C.N. (2005). Evaluation and management of laryngopharyngeal reflux. JAMA..

[10] Spantideas N., Drosou E., Bougea A., AlAbdulwahed R. (2020). Proton pump inhibitors for the treatment of laryngopharyngeal reflux. A systematic review. J Voice..

[11] Tang B., Jia L., Liu J. (2016). Clinical-psychological characteristics of refractory globus patients in China. Dig Liver Dis..

[12] Sevrain M., Brenaut E., Le Toux G., Misery L. (2016). Primary burning mouth syndrome: a questionnaire study of neuropathic and psychological components. Am J Clin Dermatol..

[13] Paterson A.J., Lamb A.B., Clifford T.J., Lamey P.J. (1995). Burning mouth syndrome: the relationship between the HAD scale and parafunctional habits. J Oral Pathol Med..

[14] Sardella A., Lodi G., Demarosi F., Uglietti D., Carrassi A. (2006). Causative or precipitating aspects of burning mouth syndrome: a case-control study. J Oral Pathol Med..

[15] Lamey P.J., Lamb A.B. (1989). The usefulness of the HAD scale in assessing anxiety and depression in patients with burning mouth syndrome. Oral Surg Oral Med Oral Pathol..

[16] Zigmond A.S., Snaith R.P. (1983). The hospital anxiety and depression scale. Acta Psychiatr Scand..

[17] Aben I., Verhey F., Lousberg R., Lodder J., Honig A. (2002). Validity of the beck depression inventory, hospital anxiety and depression scale, SCL-90, and hamilton depression rating scale as screening instruments for depression in stroke patients. Psychosomatics..

[18] Bjelland I., Dahl A.A., Haug T.T., Neckelmann D. (2002). The validity of the Hospital Anxiety and Depression Scale. An updated literature review. J Psychosom Res..

[19] Herrmann C. (1997). International experiences with the Hospital Anxiety and Depression Scale-a review of validation data and clinical results. J Psychosom Res.

[20] Goldberg D. (2006). The "NICE Guideline" on the treatment of depression. Epidemiol Psichiatr Soc..

[21] Bailey R. (2024). Why health anxiety needs NICE clinical guidelines. Lancet Psychiatry..

[22] Rim S.J., Hahm B.J., Seong S.J. (2023). Prevalence of Mental Disorders and Associated Factors in Korean Adults: National Mental Health Survey of Korea 2021. Psychiatry Investig..

[23] Lee H., Choi D., Lee J.J. (2022). Depression, anxiety, and stress in Korean general population during the COVID-19 pandemic. Epidemiol Health..

[24] Jimenez-Labaig P., Aymerich C., Braña I. (2024). A comprehensive examination of mental health in patients with head and neck cancer: systematic review and meta-analysis. JNCI Cancer Spectr..

[25] Niedzwiedz C.L., Knifton L., Robb K.A., Katikireddi S.V., Smith D.J. (2019). Depression and anxiety among people living with and beyond cancer: a growing clinical and research priority. BMC Cancer..

[26] Mitchell A.J., Chan M., Bhatti H. (2011). Prevalence of depression, anxiety, and adjustment disorder in oncological, haematological, and palliative-care settings: a meta-analysis of 94 interview-based studies. Lancet Oncol..

